# Chrysanthemum *CmDOF2* Positively Regulates Salt Tolerance in Transgenic *Arabidopsis thaliana*

**DOI:** 10.3390/plants15060936

**Published:** 2026-03-18

**Authors:** Peiling Li, Tingting Xiong, Jianhua Yue, Xinran Chong, Hanbing Xu, Zhiyong Wang, Xiang Huang

**Affiliations:** 1 College of Horticulture, Xinyang Agriculture and Forestry University, Xinyang 464000, China; 2 Jiangsu Key Laboratory for the Research and Utilization of Plant Resources, Institute of Botany, Jiangsu Province and Chinese Academy of Sciences, Nanjing Botanical Garden Mem. Sun Yat-Sen, Nanjing 210014, China

**Keywords:** *Chrysanthemum morifolium*, salt stress, CmDOF2, *Arabidopsis thaliana*

## Abstract

Chrysanthemum (*Chrysanthemum morifolium*) is a globally significant ornamental plant, whose growth, development, and ornamental quality are frequently impaired by salt stress. DOF (DNA-binding with one finger) family transcription factors extensively act as crucial regulators in medicating reactions to environmental pressures on plants. But their specific functions in regulating salt stress tolerance in chrysanthemum still remain largely elusive and require further investigation. Here, we isolated *CmDOF2*, a DOF family transcription factor from chrysanthemum, whose expression was up-regulated in chrysanthemums under salt stress. Functional analysis demonstrated that CmDOF2 functions as a nuclear-localized transcriptional activator. Comprehensive phenotypic and physiological characterization showed that heterologous expression of *CmDOF2* in *Arabidopsis thaliana* conferred markedly increased salt stress tolerance, as reflected by higher chlorophyll, leaf relative water, and proline content; lower leaf relative electric conductivity and malondialdehyde content; and increased activities of superoxide dismutase, peroxidase, and catalase. Furthermore, qRT-PCR analyses confirmed that stable expression of *CmDOF2* in *Arabidopsis* led to increased transcript levels of key salt-responsive genes, including stress marker genes (*AtRD29A*, *AtRD29B*), components of the SOS signaling pathway (*AtSOS1*, *AtSOS2*, *AtSOS3*), genes involved in osmotic adjustment (*AtP5CS1*, *AtP5CS2*), and genes encoding antioxidant enzymes (*AtSOD1*, *AtPOD34*, *AtCAT3*). Collectively, our data demonstrate that *CmDOF2* serves as a nuclear-localized transcriptional regulator with activation activity and positively regulates salt stress tolerance by mediating the transcript levels of stress-related genes in multiple signaling pathways.

## 1. Introduction

As a significant global environmental issue, soil salinization is closely linked to land degradation and ecological deterioration [1,2]. As a critical abiotic constraint, salinity dramatically inhibits plant growth, development, and productivity, even resulting in plant death under extreme conditions [3,4]. Elevated salinity triggers a series of adverse impacts on plants, including ionic toxicity, osmotic stress, and oxidative stress [5,6]. Plants initiate a variety of salt stress-related signal transduction pathways to cope with adverse environmental circumstances [7]. These pathways act either independently or synergistically, enabling plants to acclimate to and tolerate high-salt conditions [8]. Therefore, dissecting the molecular machinery underlying plant salt stress adaptation is critical for identifying promising putative genes applicable for improving salt tolerance.

Among these mechanisms, the conserved salt overly sensitive (SOS) signaling pathway, which is rapidly activated upon salt treatment, serves as a pivotal example. This pathway functions to maintain cellular ion homeostasis and consists of the SOS3, SOS2, and SOS1 proteins [9,10]. In addition, multiple transcription factor families are strongly induced by salt stress, including DREB (dehydration-responsive element binding protein), AREB (ABA responsive element binding protein), bZIP (basic leucine zipper protein), MYC (myelocytomatosis transcription factor), MYB (myeloblastosis transcription factor), WRKY, and DOF (DNA-binding with one finger) members [11,12,13,14,15,16,17]. These transcription factors mediate plant adaptation and acclimation to salinity, primarily by modulating transcriptional processes of downstream target genes [18]. Despite these advances, knowledge of the core regulatory mechanisms that underlie plant responses to salt stress still remains limited.

As a specialized group of plant-exclusive transcription factors, the DOF gene family features an extremely conserved DOF domain harboring a C2C2-type zinc finger motif, which confers the specific binding activity toward the 5′-AAAG-3′ cis-regulatory element [19]. Previous studies have shown that DOF proteins are critical for multiple physiological processes during plant growth and development, including carbohydrate metabolism [20], fruit ripening [21], flowering regulation [22], germination [23], and plant height [24]. Beyond their roles during plant development, members of the DOF family also serve pivotal functions in mediating phytohormone signaling pathways and adaptive responses to various environmental stresses [18,25,26,27]. For instance, in salt stress response, *AtDOF5.8* from *Arabidopsis thaliana* regulates the transcript level of *ANAC069* and functions in salt stress-triggered signaling pathways [28], while *ThDOF1.4* from *Tamarix hispida* enhances plant tolerance to salinity and osmotic challenges through promoting the accumulation of proline and strengthening ROS (reactive oxygen species) detoxification [29]. Studies have shown that the transcriptional expression levels of *CgDof03*, *22*, *27*, *08*, and *23* were markedly up-regulated by heat stress, suggesting their significant potential in resisting high temperatures [30]. Meanwhile, six *PpDofs* in *Prunus persica* (*PpDof1*, *8*, *9*, *15*, *16*, and *25*) were remarkably induced under low-temperature stress [31], implying their vital functions in conferring tolerance to diverse abiotic stresses. Furthermore, transient expression analysis of *CiDof22* from *Carya illinoinensis* in *Nicotiana benthamiana* showed that *NbCAT3*, *NbDreb2a*, *NbPDH1*, and *NbSOS1* (drought-associated genes) were markedly increased under 10% PEG6000 treatment, suggesting its significant responsiveness to drought stress [32]. Overexpression of *GmDof63* in soybean seedlings exhibited increased resistance against *Phytophthora sojae* by inducing the transcriptional expression of *PR1a*, *PR4*, *PR5a*, and *PR10* (pathogenesis-related protein genes), suggesting that *GmDof63* mediates the response of soybean against *P. sojae* infection by directly or indirectly regulating *PR* gene expression [33]. In summary, these results demonstrate that DOF proteins serve as critical regulators, not only in orchestrating the growth and development of the plant, but also in governing adaptive reactions to diverse abiotic stress conditions.

As a globally important horticultural species, chrysanthemum (*Chrysanthemum morifolium*) possesses significant ornamental, cultural and economic importance [34]. Salinity stress acts as a critical constraint influencing the yield and quality of chrysanthemum, often causing severe leaf chlorosis, growth retardation, and even plant mortality under severe conditions. Consequently, breeding chrysanthemum with enhanced salt tolerance is critical for stable yield and sustainable production. Previous transcriptome profiling investigations have suggested that *CmDOF2* might be involved in mediating chrysanthemum salt tolerance [35].

In the present study, we screened and characterized *CmDOF2*, a DOF gene in chrysanthemum, whose transcription was markedly induced by salt stress. Transgenic *Arabidopsis thaliana* with heterologous expression of *CmDOF2* showed significantly enhanced salt tolerance. Collectively, our findings identify *CmDOF2* as a valuable candidate for engineering enhanced salt resistance in chrysanthemum.

## 2. Results

### 2.1. Isolation and Sequence Characterization of CmDOF2

**Figure 1:**
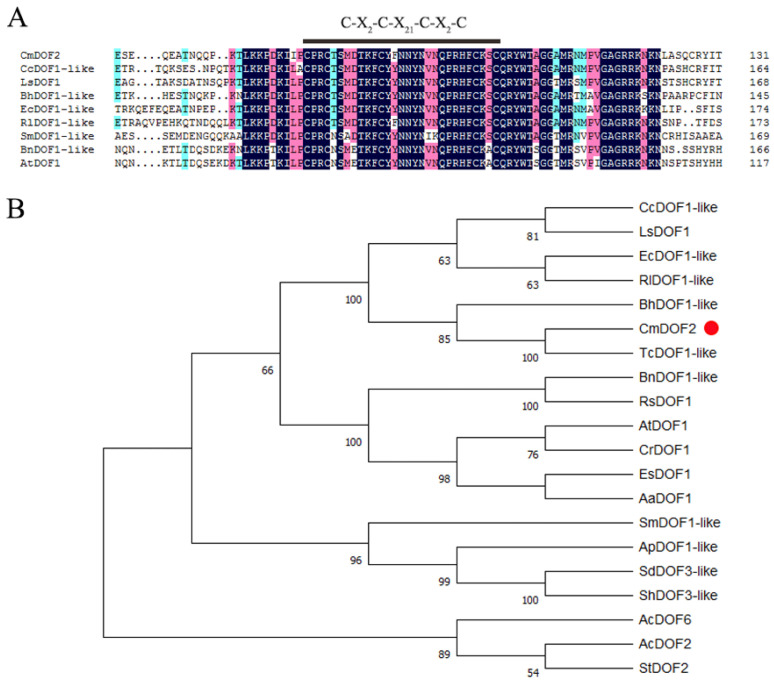
Amino acid sequence characterization and phylogenetic analysis of CmDOF2. (**A**) Alignment of amino acid sequence of CmDOF2. (**B**) Phylogenetic analysis of CmDOF2. The protein sequences used in this experiment have the following accession numbers: TcDOF1-like: GEV02235.1; CcDOF1-like: XP_024976751.1; LsDOF1: XP_023761283.1; BhDOF1-like: XP_076902525.1; EcDOF1-like: XP_043639975.1; RlDOF1-like: XP_071702357.1; SmDOF1-like: XP_057792156.1; BnDOF1-like: XP_048629735.1; AtDOF1: NP_201049.3; EsDOF1: XP_006394394.1; AaDOF1: KFK27970.1; CrDOF1: XP_006280864.1; RsDOF1: XP_056844251.1; SdDOF3-like: KAL1541631.1; AcDOF2: KAK1307898.1; StDOF2: KAF7820521.1; AcDOF6: ATG31845.1; ShDOF3-like: XP_047972319.1; ApDOF1-like: XP_051115619.1.

Full-length cDNA of *CmDOF2* (KT235676) was obtained from the chrysanthemum cultivar ‘Jinba’ based on the gene ID Unigene36536_All obtained from *C. morifolium* transcriptome data [36]. The full-length gene sequence measured 1139 bp, with an 858 bp open reading frame (ORF) which corresponds to 286 amino acid residues. Amino acid sequence analysis indicated that CmDOF2 possessed a strongly conserved DOF domain harboring C-X_2_-C-X_21_-C-X_2_-C (Figure 1A). As shown in Figure 1B, the phylogenetic tree showed that CmDOF2 protein shared the highest sequence identity with TcDOF1-like protein from *Tanacetum cinerariifolium*.

### 2.2. Subcellular Localization Analysis of CmDOF2

**Figure 2:**
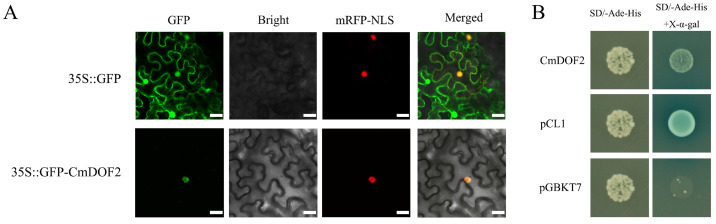
Subcellular localization and transactivation activity in CmDOF2. (**A**) Characterization of CmDOF2 subcellular localization. GFP: fluorescence signal of green fluorescent protein; mRFP-NLS: fluorescence signal of nuclear localization signal-tagged red fluorescent protein. Bars: 25 μm. (**B**) Transcriptional activity of CmDOF2.

The 35S::GFP and the 35S::GFP-CmDOF2 recombinant plasmid were separately co-transformed with nuclear marker 35S::D53-RFP into *Nicotiana benthamiana* leaf epidermal cells, to determine subcellular localization of CmDOF2. GFP fluorescence was observed exclusively in the nucleus of tobacco cells transfected with 35S::GFP-CmDOF2. By contrast, in cells transfected with the control vector 35S::GFP, the fluorescence signals of GFP were found to localize in both the nucleus and the cytoplasm (Figure 2A). Taken together, our findings showed that CmDOF2 functions as a nuclear-targeted regulator.

### 2.3. Transcriptional Activity of CmDOF2

A yeast one-hybrid system was performed to assess the transcriptional activity of CmDOF2. Only Y2H Gold yeast carrying pGBKT7-CmDOF2 or pCL1 (positive control) could grow on SD/-Ade/-His double-deficient medium, whereas yeast harboring pGBKT7 (negative control) failed to grow (Figure 2B). These results verified that CmDOF2 acts as an activator of transcription in yeast cells.

### 2.4. Expression Patterns of CmDOF2 Under Salt Treatment and in Various Tissues

**Figure 3:**
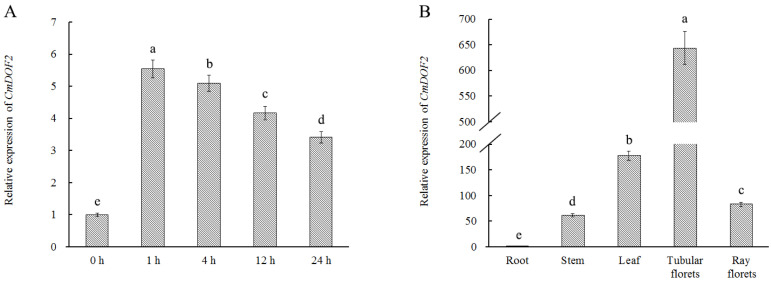
The expression profiles of *CmDOF2* in chrysanthemum. (**A**) Expression patterns of *CmDOF2* under salinity treatment. (**B**) Tissue-specific expression analysis of *CmDOF2*. Values are shown as the mean ± standard error (SE). Different lowercase letters above the columns represent statistically significant differences according to Duncan’s test.

*CmDOF2* expression patterns in various organs of ‘Jinba’ chrysanthemum and under conditions of salt stress were quantified via qRT-PCR (quantitative real-time PCR). The expression of *CmDOF2* increased quickly, then peaked at 1 h with a 5.54-fold up-regulation after salt treatment. Subsequently, the relative expression of *CmDOF2* exhibited a gradual downward trend. Nevertheless, it still remained significantly higher than control, with a 3.41-fold increase at 24 h (Figure 3A). These data indicate that CmDOF2 may participate in regulating plant adaptation to salinity stress. Meanwhile, the expression pattern of *CmDOF2* was observed in all tissues under investigation. As shown in Figure 3B, *CmDOF2* transcripts were highly abundant in tubular florets and leaves, but the lowest expression was found in roots.

### 2.5. Heterologous Expression of CmDOF2 Enhances Salt Stress Tolerance in Transgenic Arabidopsis

**Figure 4:**
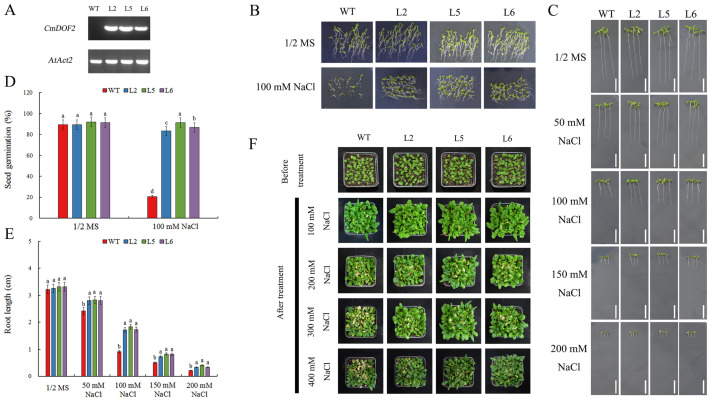
Salt stress response analysis of *CmDOF2*-expressing *Arabidopsis*. (**A**) RT-PCR-based identification of *CmDOF2*-expressing *Arabidopsis*. (**B**) Seed germination comparison between WT and *CmDOF2* transgenic lines. (**C**) Growth of WT and transgenic seedlings on 1/2 MS with NaCl. Bar: 1 cm. (**D**) Germination rates of WT and transgenic lines under salt stress. (**E**) Root length measurements of WT and transgenic lines under salt stress. (**F**) Phenotypic observations of WT and transgenic seedlings under salinity stress. Data are expressed as means ± SE. Statistical significance was analyzed by Duncan’s test and represented by distinct letters.

*Agrobacterium*-mediated transformation was employed to produce *Arabidopsis* transgenic lines expressing *CmDOF2*. Three T3 transgenic lines, L2, L5, and L6, were selected for further analysis. As shown in Figure 4A, *CmDOF2* transcripts were identified in transgenic plants, while absent in WT (wild-type) *Arabidopsis*.

Three T3 transgenic lines were assessed for their salt stress tolerance, in comparison with WT plants, focusing on seed germination, root growth, and seedling stress resistance. All seedlings germinated on 1/2 MS (half-strength Murashige and Skoog) medium exhibited no distinct phenotypic differences (Figure 4B,D). Conversely, when cultivated on 1/2 MS medium with 100 mM NaCl, three transgenic lines exhibited a markedly higher germination rate (83.33%, 91.33%, and 86.67%) than WT (20.67%).

Root length measurements demonstrated that exposure to NaCl treatment led to the inhibition of root growth in *Arabidopsis* (Figure 4D). There was no remarkable discrepancy in root length between transgenic lines and WT under 1/2 MS medium and 50 mM NaCl conditions. However, under 100 mM, 150 mM, and 200 mM NaCl conditions, transgenic lines exhibited less inhibition of root growth than WT plants. Notably, when subjected to 100 mM NaCl, WT plants showed an average root length of 0.92 cm, whereas the average values of the three transgenic lines were 1.73 cm, 1.83 cm, and 1.73 cm, respectively (Figure 4C,E).

Furthermore, three-week-old WT and transgenic seedlings were planted in soil and subjected to irrigation with NaCl solution at 100, 200, 300 and 400 mM for 14 days, respectively. Under 100 mM NaCl treatment, the phenotypic differences between WT and transgenic seedlings were marginal, accompanied by only slight leaf margin yellowing. By contrast, exposure to 200, 300, and 400 mM NaCl led to a striking phenotypic divergence between the two genotypes. WT plants exhibited severe chlorosis and wilting, reflecting greater salt sensitivity, whereas transgenic lines maintained markedly better growth (Figure 4F). Importantly, the genotypic difference in salt tolerance was further amplified with increasing NaCl concentration.

**Figure 5:**
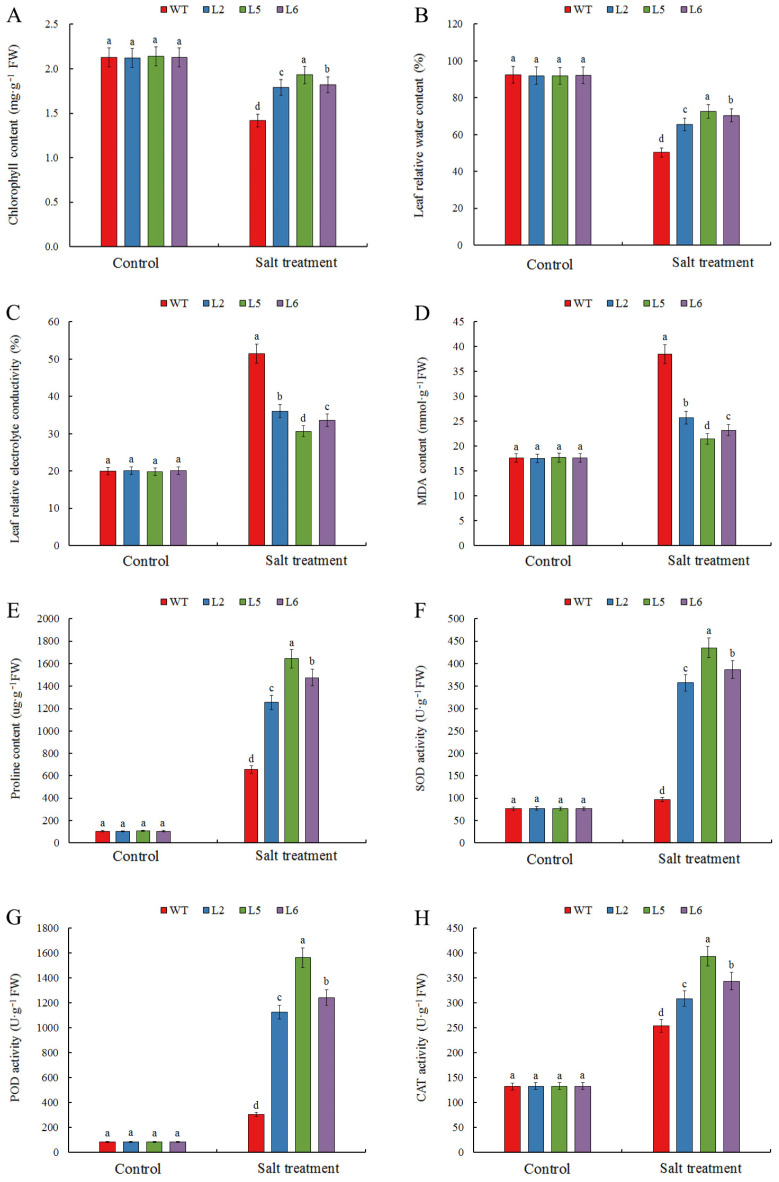
Physiological characteristics of *CmDOF2*-expressing transgenic *Arabidopsis* and WT under salt stress. (**A**) Chlorophyll content; (**B**) leaf relative water content; (**C**) leaf relative electrolyte conductivity; (**D**) MDA content; (**E**) proline content; (**F**) SOD activity; (**G**) POD activity; (**H**) CAT activity. Values are presented as means ± SE. Statistical analysis by Duncan’s test revealed significant differences, as denoted by distinct letters.

To determine whether *CmDOF2* enhances salt tolerance in *Arabidopsis*, WT and *CmDOF2* transgenic seedlings were exposed to 200 mM NaCl for 7 days. No obvious variations were observed in the physiological indicators between transgenic lines and the WT under non-stress conditions, including chlorophyll content, leaf relative water content (RWC), leaf relative electric conductivity (REC), the content of malondialdehyde (MDA) and proline, enzyme activities of superoxide dismutase (SOD), peroxidase (POD), and catalase (CAT). However, after salt stress treatment, *CmDOF2* transgenic seedlings exhibited lower REC and MDA content but higher chlorophyll content, RWC, proline content, and enzymatic activities (SOD, POD, and CAT), compared with the WT, especially transgenic line L5 (Figure 5). These results demonstrate that heterologous expression of *CmDOF2* confers significantly improved salinity tolerance in *Arabidopsis*.

### 2.6. Expression Analysis of Stress-Responsive Genes in CmDOF2-Expressing Arabidopsis

**Figure 6:**
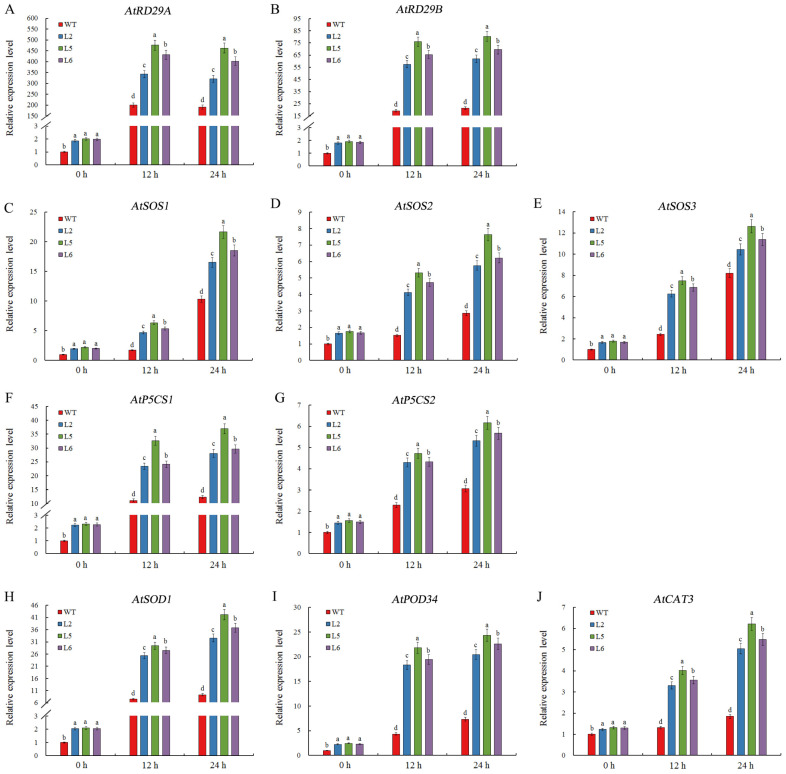
Transcript levels of stress-related genes in *CmDOF2*-expressing *Arabidopsis* and WT under salt treatment. (**A**) *AtRD29A*; (**B**) *AtRD29B*; (**C**) *AtSOS1*; (**D**) *AtSOS2*; (**E**) *AtSOS3*; (**F**) *AtP5CS1*; (**G**) *AtP5CS2*; (**H**) *AtSOD1*; (**I**) *AtPOD34*; (**J**) *AtCAT3*. Values represent the means ± SE. Distinct letters represent statistically significant variation between transgenic lines and WT by Duncan’s test.

To further explore the potential role of *CmDOF2* under salt stress, the relative transcript abundances of multiple salt-responsive genes were assessed in both *CmDOF2* transgenic lines and the WT. Transcript levels of stress-marker genes (*AtRD29A* and *AtRD29B*), SOS pathway genes (*AtSOS1*, *AtSOS2*, and *AtSOS3*), osmotic adjustment-related genes (*AtP5CS1* and *AtP5CS2*), and antioxidant enzymes-encoding genes (*AtSOD1*, *AtPOD34*, and *AtCAT3*) were determined in *CmDOF2* transgenic lines and the WT upon salt stress (Figure 6). Under non-stress conditions (0 h, without salinity treatment), the transcript abundance of *AtPOD34* was remarkably higher in *CmDOF2* transgenic lines than the WT, whereas the remaining nine genes exhibited analogous transcript expression profiles between the two groups. When subjected to salinity stress, all ten tested genes exhibited higher expression levels in *CmDOF2*-expressing lines than the WT. The data demonstrate that *CmDOF2* improves salt resistance in *Arabidopsis* though regulating a range of signaling pathways.

## 3. Discussion

Plants have evolved diverse signaling pathways to perceive and respond to environmental stresses, among which transcription factors act as crucial regulators [37]. Ever since *ZmDof1* was isolated and characterized in 1993 as the first DOF protein in maize [38], this family has been successively characterized in an increasing number of plant species [32,39,40,41,42,43,44,45,46]. As key regulatory proteins, DOF transcription factors participate extensively during various biological events, including plant growth, development, and adaptation to stresses [18,47].

To date, 20 DOF proteins have been reported from chrysanthemum [35], yet systematic investigations into their precise biological functions remain largely limited. As a DOF family protein in plants, *CmDOF6* regulates chrysanthemum plant height by suppressing *CmGA20ox1* expression via the mediation of CmTCP8 [24]. In this study, CmDOF2 was found to harbor a complete C2C2-type single zinc finger domain, and to function as a nuclear-localized transcriptional activator (Figure 1A and Figure 2), matching the typical structural and functional traits of the DOF family [19]. Notably, *CmDOF2* transcripts were found to accumulate in chrysanthemum tissues, and its transcript level was markedly induced under salt stress (Figure 3), similar to the four DOF genes from *Camellia oleifera* (*ColDof1*, *ColDof2*, *ColDof14*, and *ColDof36*) [48]. Collectively, our results indicate that *CmDOF2* probably participates in mediating chrysanthemum’s response to salinity stress.

To better understand how *CmDOF2* regulates salt stress response at the molecular level, *Arabidopsis thaliana* transgenic lines ectopically expressing *CmDOF2* were successfully obtained. Under salinity stress conditions, transgenic lines expressing *CmDOF2* exhibited notably higher seed germination rate and longer root length than the WT (Figure 4B–E). Our results agree with previous findings reported for *GhDof1.7* [49]. Moreover, when exposed to salt stress with different NaCl concentrations, *CmDOF2* transgenic lines exhibited better phenotypic performance than WT plants. (Figure 4F).

When exposed to adverse conditions, variations in plant physiological indices mirror plant metabolic status and adequately demonstrate the strength of plant stress tolerance [4,7]. Leaf chlorophyll content serves as a crucial physiological parameter positively associated with plant photosynthetic capacity [50], while RWC is frequently used as an indicator reflecting both plant water status and tissue water metabolism [51]. Previous studies have reported that leaf chlorophyll content and RWC often decrease under salt stress, and plants displaying a milder decrease in these parameters are widely recognized to possess greater salt tolerance [52,53]. In this study, compared with the WT under salt stress treatment, *CmDOF2*-expressing transgenic plants displayed greater chlorophyll content and RWC (Figure 5A,B), suggesting that heterologous expression of *CmDOF2* confers increased plant tolerance to salt stress. ROS can induce cellular membrane lipid degradation and exacerbate lipid peroxidation under salt stress. Cell membrane disruption causes a rise in REC [54,55]. Meanwhile, MDA content is extensively used as a stable indicator to assess the extent of oxidative damage to membrane lipids [56,57]. In our study, under salt stress with 200 mM salt solution, *CmDOF2*-expressing transgenic lines displayed relatively higher REC and MDA content, whereas WT plants exhibited lower levels of these two indicators (Figure 5C,D).

Under stress conditions, plants can accumulate osmotic regulators such as proline, which effectively regulate cellular osmotic potential and enhance cell water retention capacity, and thereby maintain normal cellular physiological and biochemical metabolism [58,59]. In the present study, when subjected to salt stress, *CmDOF*2-expressing transgenic lines accumulated greater proline accumulation compared with the WT (Figure 5E), suggesting that *CmDOF2* might enhance the osmotic adjustment capacity of transgenic plants by positively regulating proline anabolism under salt stress. Salt stress in plants also can trigger excessive ROS accumulation [1,3]. To mitigate oxidative damage, plants form an enzymatic antioxidant defense system by synthesizing vital antioxidant enzymes such as SOD, POD, and CAT [60,61]. The antioxidant enzymes scavenge excess ROS and reduce membrane lipid peroxidation, thus improving salt resistance in plants [62,63]. In our current work, markedly elevated SOD, POD, and CAT activities in *CmDOF2*-expressing transgenic plants were exhibited in comparison with the WT when exposed to salt treatment (Figure 5F–H). These findings indicated that *CmDOF2* could effectively scavenge the accumulation of intracellular ROS by strengthening antioxidant enzyme activities, thereby relieving oxidative injury triggered by salt stress.

Furthermore, the expression of multiple genes, including stress-responsive genes, genes associated with proline metabolism, and antioxidant enzyme biosynthesis, were determined by qRT-PCR. Previous studies have demonstrated that *AtRD29A* and *AtRD29B* participate in stress-induced detoxification and alleviate stress-induced damage in plants [64,65]. Typical SOS pathway-related genes in plants, *AtSOS1*, *AtSOS2*, and *AtSOS3*, are essential for sustaining ion homeostasis and salt resistance [10,66]. *AtP5CS1* and *AtP5CS2* participate in proline biosynthesis and accumulation under saline conditions [67,68]. Synergistic regulation of the antioxidant enzyme system constitutes a central strategy for plants to counteract oxidative damage, in which *AtSOD1* encodes the cytosolic copper/zinc superoxide dismutase CSD1 for scavenging superoxide radicals, *AtPOD34* may participate in the production of H_2_O_2_, and *AtCAT3* encodes a catalase that facilitates the conversion of H_2_O_2_ into molecular oxygen and water [69,70,71]. In our study, under salt stress, stress-related genes exhibited stronger induction in *CmDOF2*-expressing lines than the WT (Figure 6), suggesting that *CmDOF2* improves salt resistance via activating the transcript levels of stress- associated genes.

In summary, *CmDOF2*, a member belonging to the DOF gene family, is markedly induced by salt stress in chrysanthemum. *CmDOF2*, a nucleus-localized transcriptional activator, positively regulates salt tolerance in transgenic *Arabidopsis*. These findings indicate that CmDOF2 positively modulates expression of stress-associated genes to improve salt resistance, probably by modulating osmoprotectant accumulation and the function of the antioxidant enzyme system. However, the mechanistic details of *CmDOF2*-mediated signaling pathways underlying stress responses in chrysanthemum plants has not yet been fully clarified. In further studies, the generation of transgenic chrysanthemum plants overexpressing *CmDOF2* will contribute to uncovering the detailed regulatory molecular mechanisms by which *CmDOF2* regulates salt stress tolerance in plant.

## 4. Materials and Methods

### 4.1. Chrysanthemum Growth Conditions and Salt Treatment

The experimental materials used for the present study were chrysanthemum cv. ‘Jinba’. The chrysanthemum seedlings were planted in pots containing a (1:1, *v*/*v*) peat-vermiculite mixture and incubated in the plant growth chamber. Control growth conditions were set as follows: a 16/8 h light/dark regime, 22 ± 1 °C, 70% relative humidity, and a light intensity of 100 μmol·m^−2^·s^−1^.

Tissue samples of chrysanthemum, including roots, stems, leaves, tubular florets, and ray florets were separately harvested from plants for total RNA extraction. For salt stress, plants were treated with 200 mM NaCl at stages 7–9 [72], then the second fully expanded leaves were harvested at 0 h, 1 h, 4 h, 12 h, and 24 h after treatment. Then leaves were frozen instantly in liquid nitrogen and preserved at −80 °C for further experiments.

### 4.2. Isolation and Sequence Analyses of CmDOF2

Total RNA was isolated from chrysanthemum ‘Jinba’ leaf samples with the RNAiso reagent (TaKaRa, Dalian, China). cDNA was then prepared using M-MLV reverse transcriptase (TaKaRa, Dalian, China). The full-length cDNA sequence of *CmDOF2* was amplified by PCR using primers CmDOF2-F/R (Table S1). After that, the PCR products were transferred into pMD19-T for subsequent sequencing. Homologous polypeptide sequences of CmDOF2 from various plant species were obtained via an online BLAST search (https://blast.ncbi.nlm.nih.gov/Blast.cgi, accessed on 3 March 2023). DNAMAN 6.0 software (Lynnon Biosoft, San Ramon, CA, USA) was utilized to perform amino acid sequence comparison of CmDOF2 and its homologs. Phylogenetic analysis was performed using MEGA 11 software [73] with the neighbor-joining (NJ) algorithm, p-distance model, and 1000 bootstrap replicates.

### 4.3. Plasmid Construction and Arabidopsis thaliana Transformation

The coding sequence (CDS) of *CmDOF2* was initially amplified by PCR using the specific primer pair CmDOF2-ENTR-F/R (Table S1) at *Sal* I and *Not* I restriction sites, and subsequently ligated into the pENTR™ 1A Gateway vector (Invitrogen™, Thermo Fisher Scientific, Waltham, MA, USA). This recombinant entry vector was subsequently introduced into the overexpression vector pMDC43 (35S::GFP) via recombination to generate the plasmid pMDC43-CmDOF2 (35S::GFP-CmDOF2).

The recombinant construct pMDC43-CmDOF2 was introduced into the *Agrobacterium tumefaciens* strain GV3010, then transferred into wild-type *Arabidopsis thaliana* (WT) through the floral dipping technique [74]. Independent transgenic lines were selected on hygromycin-supplemented MS medium at 20 µg·mL^−1^. The CmDOF2-RT-F/R primer pair (Table S1) was used to identify T_3_ generation plants via RT-PCR, and the *AtACT2* gene (AT3G18780.2) was used as the internal reference.

### 4.4. Subcellular Localization of CmDOF2

Additionally, 5-week-old *Nicotiana benthamiana* seedlings were used for co-infiltration with *Agrobacterium tumefaciens* harboring either the 35S::GFP-CmDOF2 or the 35S::GFP (negative control), together with the p19 strain, according to a previously published protocol [75]. The 35S::D53-RFP vector was employed as a marker for nuclear localization in this experiment [76]. After incubation in dark conditions at 22 °C for 48 h, GFP fluorescence signals were examined using the TCS SP8 laser confocal scanning microscope (Leica, Wetzlar, Germany).

### 4.5. Transcriptional Activation Analysis of CmDOF2

PCR was used to obtain the CDS of *CmDOF2* (without termination codon) using the specific primer pair CmDOF2-BD-F/R (Table S1), then ligated into the pGBKT7 vector at *Eco*R I and *Bam*H I sites. The yeast strain Y2H was transformed with pGBKT7-CmDOF2 constructed plasmid, pCL1 (positive control), and pGBKT7 (negative control), following the manufacturer’s protocol. The yeast cells harboring pCL1 plasmid were propagated on SD/-Leu medium, while yeast transformed with pGBKT7-CmDOF2 and pGBKT7 was separately cultured on SD/-Trp medium. The obtained colonies were then inoculated onto SD/-His-Ade selective media, with one group supplemented with 40 mg·L^−1^ X-α-gal. The transcriptional activation activity was assessed after all samples were incubated at 30 °C for 72 h.

### 4.6. Salt Stress Treatment on Transgenic Arabidopsis Plants

To assess seed germination, 50 seeds from *CmDOF2* transgenic *Arabidopsis* and WT were sown and germinated on 1/2 MS agar medium supplemented with or without 100 mM NaCl. The cotyledon greening rate was calculated and statistically analyzed after 7 days of cultivation. To determine primary root elongation, seeds were initially placed on 1/2 MS medium for germination over 3 days, then transferred to fresh 1/2 MS medium with gradient NaCl concentrations (0, 50, 100, 150, 200 mM) for another 7 days, after which primary root length was measured. In addition, three-week-old transgenic plants expressing *CmDOF2* and WT plants were subjected with NaCl solution at various concentrations (100, 200, 300, and 400 mM), respectively. After 14 days of salt stress, plant phenotypes characteristics were photographed to facilitate subsequent analysis. Furthermore, three-week-old WT and *CmDOF2* transgenic seedlings were treated with 200 mM NaCl solution. After 7 days of treatment, leaves were harvested to determine physiological indexes. Meanwhile, leaves were collected at 0, 12, and 24 h under salinity treatment to analyze the expression of stress-related genes.

### 4.7. Determination of Physiological Indexes of Arabidopsis

The chlorophyll contents of leaves were determined using 80% acetone extraction [77]. RWC was determined following the previously described method [78]. REC was measured using the P902 electric conductivity meter (Youke, Shanghai, China) to estimate leaf cell membrane stability, as described by Su et al. [79]. The contents of MDA and proline, along with enzymatic activities of SOD, POD, and CAT, were measured using corresponding commercial assay kits. All the kits used were procured from the Nanjing Jiancheng Institute of Biological Engineering (Nanjing, China), with detailed information as follows: MDA assay kit (Cat. No. A003-1); proline assay kit (Cat. No. A107-1-1); SOD assay kit (Cat. No. A001-3) assayed by the WST-1 method; POD assay kit (Cat. No. A084-3-1); CAT assay kit (Cat. No. A007-1-1) analyzed via the ammonium molybdate method.

### 4.8. qRT-PCR Analyses

qRT-PCR assays were executed on a 7500 Fast Real-Time PCR System (Thermo Fisher Scientific, Waltham, MA, USA), using SYBR^®^ Premix Ex Taq^TM^ II (Tli RNaseH Plus) purchased from TaKaRa (Dalian, China). Each sample was subjected to triplicate biological and technical repetitions during analysis. Relative expression was calculated by the 2^−ΔΔCT^ approach [80]. For chrysanthemum and *Arabidopsis* samples, the *CmEF1α* gene (KF305681) and *AtACT2* (AT3G18780.2) were employed as reference genes, respectively. To investigate the expression of salt stress-responsive genes in *CmDOF2* transgenic and WT plants, the following genes were selected: *AtRD29A* (AT5G52310.1), *AtRD29B* (AT5G52300.1), *AtSOS1* (AT2G01980.1), *AtSOS2* (AT5G35410.1), *AtSOS3* (AT5G24270.1), *AtP5CS1* (AT2G39800.1), *AtP5CS2* (AT3G55610.1), *AtSOD1* (AT1G08830.1), *AtPOD34* (AT3G49120.1), and *AtCAT3* (AT1G20620.1). The sequences of all related primers are provided in Table S1.

### 4.9. Statistical Analysis

Statistical analyses were performed using SPSS v17.0 (SPSS Inc., Chicago, IL, USA). Duncan’s test was used to evaluate trait variations, with statistical significance defined as *p* < 0.05.

## Data Availability

The original contributions presented in this study are included in the article/Supplementary Material. Further inquiries can be directed to the corresponding author.

## Supplementary Materials

The following supporting information can be downloaded at: https://www.mdpi.com/article/10.3390/plants15060936/s1, Table S1: Primer names and sequences used in this study.
